# NGSEP3: accurate variant calling across species and sequencing protocols

**DOI:** 10.1093/bioinformatics/btz275

**Published:** 2019-04-25

**Authors:** Daniel Tello, Juanita Gil, Cristian D Loaiza, John J Riascos, Nicolás Cardozo, Jorge Duitama

**Affiliations:** 1 Systems and Computing Engineering Department, Universidad de los Andes, Bogotá 111711, Colombia; 2 Biotechnology lab, Centro de Investigación de la caña de azúcar de Colombia, CENICAÑA, Cali 760046, Colombia; 3 Agrobiodiversity Research Area, International Center for Tropical Agriculture, Cali 763537, Colombia

## Abstract

**Motivation:**

Accurate detection, genotyping and downstream analysis of genomic variants from high-throughput sequencing data are fundamental features in modern production pipelines for genetic-based diagnosis in medicine or genomic selection in plant and animal breeding. Our research group maintains the Next-Generation Sequencing Experience Platform (NGSEP) as a precise, efficient and easy-to-use software solution for these features.

**Results:**

Understanding that incorrect alignments around short tandem repeats are an important source of genotyping errors, we implemented in NGSEP new algorithms for realignment and haplotype clustering of reads spanning indels and short tandem repeats. We performed extensive benchmark experiments comparing NGSEP to state-of-the-art software using real data from three sequencing protocols and four species with different distributions of repetitive elements. NGSEP consistently shows comparative accuracy and better efficiency compared to the existing solutions. We expect that this work will contribute to the continuous improvement of quality in variant calling needed for modern applications in medicine and agriculture.

**Availability and implementation:**

NGSEP is available as open source software at http://ngsep.sf.net.

**Supplementary information:**

Supplementary data are available at *Bioinformatics* online.

## 1 Introduction

High-throughput sequencing (HTS) technologies arguably represent the biggest biotechnology breakthrough in this century (Goodwin *et al.*, 2016). After their commercial introduction in 2007 their use quickly expanded from basic and applied genetics research to practice in medicine, food and energy production and preservation of biodiversity. Nowadays, both the sequencing technologies and the bioinformatic pipelines required to analyze the datasets generated by HTS technologies are well known among both researchers and practitioners in different fields.

For species having an available reference genome, common pipelines to analyze HTS reads include three main steps: (i) reads are aligned to the reference genome, (ii) variation against the reference genome is identified and (iii) the genotype of each sequenced sample is predicted for each variant identified in the second step. Steps (ii) and (iii) are often called discovery and genotyping, respectively. Discovery and genotyping may be combined in cases such as genetic testing in clinical settings in which only one individual is analyzed at a time. Conversely, discovery and genotyping are considered as two separate processes in cases in which complete populations are analyzed at a time. For example, a typical pipeline for genomic selection in plant breeding includes an initial discovery step over the base population followed by subsequent genotyping steps, first for the same base population, and later for the advanced populations developed at each step of the breeding cycle (Crossa *et al.*, 2013). Accurate genotyping is at least as important as accurate variants discovery. For genetic-based diagnosis, the effect of a potentially damaging variant will probably be very different if the related genotype is homozygous or heterozygous (Goldfeder *et al.*, 2016). For genome-wide association studies, widely performed in both human and plant genetics, accurate genotyping is critical because errors in the genotype of even a small fraction of the population can mislead true correlations between variants and phenotypes.

Widely used tools to perform variant discovery and genotyping from aligned reads include the genome analysis toolkit (GATK) (McKenna *et al.*, 2010; Poplin *et al.*, 2017), Bcftools (Li, 2011), Freebayes (Garrison and Marth, 2012), Platypus (Rimmer *et al.*, 2014), Varscan2 (Koboldt *et al.*, 2012), Strelka2 (Kim *et al.*, 2018), among others. Our research group maintains the Next-Generation Sequencing Experience Platform (NGSEP), which was originally created to perform variants discovery and genotyping, combining different algorithms to detect not only single nucleotide variants (SNVs) but also small and large indels, copy-number variants and inversions (Duitama *et al.*, 2014). The core process in all these tools, called pileup, traverses the aligned reads over the genome to identify potential variation sites and collect candidate alleles per site (Li, 2011). For each potentially variant site, NGSEP, Bcftools, Freebayes and the original implementation of GATK called the Unified Genotyper (McKenna *et al.*, 2010) implement Bayesian approaches with different likelihood functions to find the most likely genotype, taking into account the base quality scores provided by the sequencing instrument. The GATK Haplotype caller (Poplin *et al.*, 2017), Platypus and Strelka2 implement a local *de-novo* assembly and haplotype reconstruction, with the goal of improving indel discovery and genotyping. Several other works try to devise combinations of subsets of these tools to improve the overall accuracy beyond that of each individual tool (Supplementary Table S1). A recent approach called DeepVariant tries to improve accuracy by training a convolutional neural network from pileup images of candidate variant sites (Poplin *et al.*, 2018).

Previous studies show that detection and genotyping of small indels through the pileup process is more difficult than SNV discovery and genotyping (Fang *et al.*, 2014; Jiang *et al.*, 2015; Li and Wren, 2014). First, possible alleles of indel events are not known beforehand but must be discovered along the process. And second, base calling errors not only can produce differences between reads sequenced from the same indel allele, but also can produce misalignments of reads around indel sites (Tran *et al.*, 2016). These misalignments can create false indel calls, incorrect genotypes and even false SNVs in the surrounding regions. This problem is particularly evident around short tandem repeats (STRs) and especially large homopolymers (Supplementary Fig. S1). Overall this has been identified as one of the major sources of false positives for both discovery and genotyping of SNVs, indels and STRs. Other issues arise in specific protocols such as whole exome sequencing (WES) in which fragments spanning mutant alleles of sufficiently large indels may not be identified by the capture process (Fang *et al.*, 2014).

NGSEP was conceived and released as open source software in 2013 as an accurate, efficient and easy-to-use software for general variants detection (Duitama *et al.*, 2014). For version 2, we added different functionalities to facilitate downstream analysis of genotyping-by-sequencing (GBS) data from populations of hundreds of individuals including read demultiplexing, genotype imputation, allele sharing statistics and detection of haplotype introgressions (Perea *et al.*, 2016). Here we present the new algorithms implemented in NGSEP version 3, performing realignment around potential indels and known STRs to improve the accuracy of SNV and small indel detection and, at the same time, achieving accurate genotyping of the STRs themselves. This work is complemented by other functionalities released in this version including distributions of *k*-mer abundances, estimation of general and per chromosome ploidy levels, detection of haplotype introgressions, calculation of distance matrices from VCF files as well as utilities for benchmark experiments, such as simulation of single individuals from reference genomes and validation of genotype calls against a gold standard VCF file. Extensive validation experiments on both simulations and real benchmark datasets of yeast, rice, cassava and humans following widely used sequencing protocols, such as whole genome sequencing (WGS), WES and GBS, show that NGSEP has comparative accuracy and better efficiency for both discovery and genotyping of SNVs, indels and STRs in a wide variety of scenarios.

## 2 Materials and methods

### 2.1 Algorithm for STR-aware realignment and indel calling

**Fig. 1.**
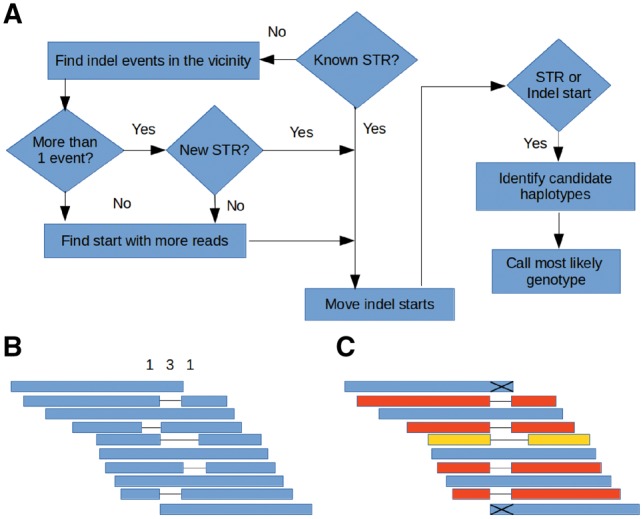
Schematic procedure for indel realignment and haplotype clustering. (**A**) Flowchart with the overview of the process. (**B**) Voting mechanism to select the most likely start of a given indel. Blue bars represent aligned reads. The numbers on top represent the reads supporting each possible start position. (**C**) Realignment of reads supporting indel events according to the results of the voting procedure and clustering of candidate haplotypes to discover and genotype small indels. Reads that do not span across the indel (marked with X) are soft-clipped

The original process implemented in NGSEP to traverse a sorted set of alignments against the reference genome and extract allele calls for each reference site (usually called pileup) completely relied on the quality of the alignments produced by the read alignment tools. To improve the accuracy of variant discovery, we implemented in NGSEP the following algorithm to realign reads spanning potential indels and STRs. After all alignments spanning one particular reference position *i* have been collected, if an indel is identified in at least one alignment starting from position *i*, the algorithm collects additional indel calls starting from the next *x* positions where *x* is the length of the largest indel event starting at position *i *+* *1. The variant discovery algorithm then runs a voting procedure to choose the position *j* with the largest number of indel starts within the collected indel calls. Once this position is chosen, alignments supporting indel starts in positions different than *j* are modified to start at position *j*. To make this process efficient, a full realignment is not recalculated but only the description of the alignment (CIGAR) is modified in memory. If i≠j, the pileup process finishes collecting allele calls only for SNV discovery, and the indel discovery is deferred to the moment when the pileup process reaches position *j*. If *i *=* j*, then alleles are collected for indel discovery. If known STRs are provided as input of the discovery process, instead of a voting mechanism to pick the most likely start of an indel event, the start position of the STR is chosen to modify alignments having indel events within the STR. The same procedure is followed for known indels and STRs during the genotyping step. Figure 1 shows a schematic of the entire procedure.

Unlike SNV discovery in which calls can only be made to at most four possible basepairs, the indel discovery procedure needs to figure out possible alleles directly from the data. Once reads are consistently aligned, the position *k* is calculated as the final position of the largest deletion call plus 1 (*i *+* *1 if only insertion calls are present), and the interval [i,k] is treated as a single locus to collect candidate haplotypes for indel discovery. To prevent false allele calls produced by dangling ends, reads that do not completely cover the interval [i−5,k+5] are soft-clipped to remove the end partially spanning the interval. For each read *r* completely covering the interval [i−5,k+5], the segment of each read aligning to the interval [i,k] is extracted and collected as a candidate haplotype supported by *r*. A first alternative to infer the real haplotypes corresponding to the analyzed interval is to treat directly the candidate haplotypes as possible alleles and select the most likely genotype following the same Bayesian model used for SNV discovery. However, as the indel event becomes larger, reads that were originally sequenced from one single haplotype can become different within the region spanning the indel due to sequencing errors. To overcome this issue, we implemented a two level clustering on these initial candidate haplotypes. First, haplotypes are clustered by length. Then, for each cluster with at least 10 elements an ungapped alignment is built, variable positions are calculated, and second level clusters are built based on Hamming distance. The Hamming consensus of each cluster is calculated and nominated as a representative haplotype. The Bayesian model is then executed using as possible alleles these representative haplotypes. The conditional probability of an allele call with the same length of a given representative haplotype is the product of success probabilities for matching basepairs and error probabilities for non-matching basepairs in an ungapped alignment between the call and the haplotype. Conversely, the conditional probability of an allele call given a representative haplotype of different length is assigned to a fixed low value representing the indel error probability (0.0001 by default).

Similar to the behavior of other tools, we implemented a new command that considers the complete set of population reads through the pileup process, including the realignment steps described above. In contrast with the traditional sample-by-sample analysis (Duitama *et al.*, 2014) this new functionality discovers variants and genotypes each individual of the population within a single step. This makes the process more efficient in running time and potentially increases the quality of the population variants (especially novel indels) because the voting procedure to realign reads is performed across reads from the entire population.

### 2.2 Real data gathering and construction of gold standard datasets

Benchmark experiments were performed using different publicly available sequencing datasets of yeast, rice, cassava and human samples. Accession IDs, sequencing protocols and URLs for downloading of the datasets and their corresponding reference genomes are available in the Supplementary Table S2. Yeast samples correspond to two haploid parental strains (CBS4C and ER7A) with contrasting glycerol/ethanol production ratio, and a pool of 20 randomly selected haploid F1 segregants from the cross of the two parental strains (Hubmann *et al.*, 2013). For humans, we downloaded publicly available read alignments from the Hapmap human individual NA12878 and from the synthetic diploid individual, for which gold standard genotype calls and confident regions were previously obtained and termed respectively the Platinum Genomes (PlatGen) dataset (Eberle *et al.*, 2017) and the SynDip dataset (Li *et al.*, 2018). We also downloaded WES data from two different capture experiments for NA12878, using respectively the SeqCap EZ human exome library v3.0 (also known as Nimblegen) and the Illumina TruSeq Exome Capture library. Finally, we analyzed public GBS data from two plant biparental populations: A cassava full-sib F1 segregating population, termed the K-family, consisting of 132 individuals derived from the cross between cultivars TMS30573 and CM2177-2 (Fregene *et al.*, 1997), which had been previously sequenced and used to generate a dense SNP-based genetic map (Soto *et al.*, 2015), and the Azucena x IR64 rice biparental population, including the two parental lines plus 171 F6 siblings (Spindel *et al.*, 2013). Both populations were sequenced using the GBS protocol, which has been widely used in plant population genetics as a cost-effective alternative to WGS (Elshire *et al.*, 2011).

Gold standard variant calls and confidence regions were already determined for the PlatGen and SynDip samples. For the yeast unselected pool, a procedure similar to that described in Duitama *et al.* (2014) but including the tools compared in this manuscript was followed to build a gold standard from variant calls predicted by the different tools from the reads of the two parental strains aligned to the reference genome using bwa (Li and Durbin, 2009) and bowtie2 (Langmead and Salzberg, 2012). In brief, a heterozygous call for the pool is predicted if at least 10 of the 12 possible combinations of alignment tool and variant caller predict a homozygous variant for one parent and at most two of them predict a variant at the same site for the other parent. A homozygous call for the pool is predicted if at least 10 of the 12 possible combinations of alignment tool and variant caller predict the same homozygous variant for both parental strains. The exact procedure is implemented as a script available with the distribution of NGSEP (class ngsep.benchmark.BiparentalHaploidGoldStandardBuilder).

### 2.3 Comparison with gold standards

Six different software tools were executed on each dataset (see Supplementary Text for execution details). To compare gold standard genotype calls with those predicted by each tool, a custom script was built and made available with NGSEP v3.3.1 (VCFGoldStandardComparator command). Similar to the approach implemented in the VarMatch software tool (Sun and Medvedev, 2017), gold standard calls are clustered based on proximity to each other or to an STR. However, instead of trying to predict STRs from the reference genome, we provide to the script STRs predicted from tandem repeats finder (Benson, 1999). Test calls within or close (<5 bp) to each cluster are collected and matched with gold standard calls. Given that variants are phased in the gold standard VCF files, true haplotypes are built for each cluster. Then, possible haplotypes are built from test calls following an exhaustive procedure for clusters with <9 heterozygous test calls and a greedy procedure for other clusters. A cluster is called true positive if a pair of possible test haplotypes matches the gold standard haplotypes, or a genotyping error if none of the test haplotype pairs can match the gold standard haplotypes. Test calls within confidence regions and outside clusters are treated as false positives. Clusters without matched test calls are treated as false negatives.

For the cassava GBS population, patterns of Mendelian inheritance and population statistics are used to categorize variants and to identify genotyping errors (Perea *et al.*, 2016). Sensitivity is calculated as the number of genotype calls in variants that can be used to build a genetic map. Genotype errors are identified as calls that are inconsistent with the category assigned to each variant. A custom script to calculate datapoints and errors for each possible filter of minimum genotype quality is available with the NGSEP distribution (class ngsep.benchmark.QualityStatisticsOutbredF1Families). A similar procedure was implemented for the rice biparental family but taking into account that the population should have low heterozygosity due to inbreeding. Sensitivity is calculated as the number of genotype calls in variants in which the two parents are homozygous for different alleles, have a minor allele frequency >0.1, and an expected heterozygosity <0.1. Genotype errors are identified as genotype calls having the minor allele [homozygous or heterozygous] in variants with minor allele frequency <0.1 or heterozygous calls within the variants considered for sensitivity. The exact procedure is available with the NGSEP distribution (class ngsep.benchmark.QualityStatisticsInbredBiparentalFamilies).

## 3 Benchmark experiments

### 3.1 Yeast unselected pool

**Fig. 2.**
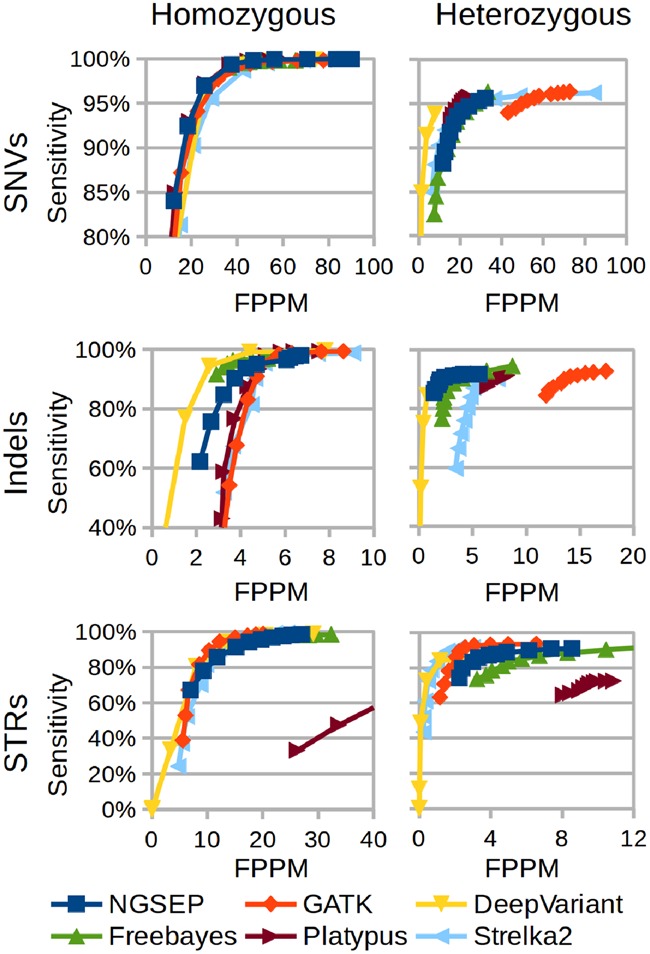
Comparison of different tools for variants discovery from reads taken from an F1 pool of segregants derived from two yeast haploid strains. Results are discriminated by variant type (SNVs, Indels and STRs) and gold standard genotype (homozygous variant or heterozygous). The proportion of FPPM is used as a measure of specificity. Curves are obtained varying the filter of minimum genotype quality (GQ field in the VCF file) from 0 to 90

As a first step to assess the performance of NGSEP using real datasets, we updated the benchmark experiment with a yeast F1 unselected pool described in Duitama *et al.* (2014). NGSEP was compared with the GATK haplotype caller (Poplin *et al.*, 2017), Bcftools (Li, 2011), Freebayes (Garrison and Marth, 2012), Platypus (Rimmer *et al.*, 2014), Strelka2 (Kim *et al.*, 2018) and DeepVariant (Poplin *et al.*, 2018). In brief, in this experiment two yeast haploid parentals were sequenced at high read depth (> 80×). Hence, accurate homozygous and heterozygous sites in a pool of unselected F1 segregants can be accurately predicted from consensus of haploid parental genotype calls. True and false positives and negatives are calculated separately for homozygous and heterozygous sites, and for SNVs, indels and STRs. As recently suggested (Li *et al.*, 2018), we used the false positives per million basepair (FPPM) as a measure of specificity. Instead of applying commonly used filters on read depth or strand bias, we examined the effect of relying on the quality score reported by each tool (GQ field in the VCF files) as the main predictor of quality for each genotype call. Figure 2 and Supplementary Figure S2 show the overall results of this experiment as receiving operator characteristic like curves obtained varying the minimum GQ value (exact values of sensitivity and FPPM are available in Supplementary Table S3). Increasing stringency by raising the minimum GQ value produces an important reduction of FPPM values for all tools and variant types. For example, FPPM values reported by NGSEP and GATK for homozygous SNVs can be reduced up to 50 points with <2% loss of sensitivity. A similar trend is observed in heterozygous SNVs reported by Strelka2. Filtering using GQ values reduces sensitivity in more than 10% for all variant types reported by DeepVariant and Strelka2 and for homozygous variants reported by NGSEP, GATK and Platypus. Freebayes has the smallest reduction of sensitivity (about 5% for homozygous variants and <18% for heterozygous variants). Comparing accuracy between tools, the most noticeable differences are a much lower accuracy of Bcftools for indels (Supplementary Fig. S2) and of Platypus for STRs. GATK and Strelka2 show higher accuracy than NGSEP for STRs but NGSEP is more accurate for SNVs and indels. DeepVariant shows the best accuracy for heterozygous SNVs and homozygous indels before genotype quality filters. The accuracy of Freebayes is similar to that of NGSEP for SNVs and indels but the FPPM values of Freebayes are larger than those of NGSEP for STRs. We checked, comparing with version 2.1.3 of NGSEP, that the realignment and allele clustering algorithms presented in this work effectively improve accuracy for all variant types (Supplementary Fig. S2). Comparing accuracy between variant types, the sensitivity to detect indels and STRs is between 5 and 20% lower than that of SNVs. The FPPM values of homozygous SNVs were similar to those of heterozygous SNVs. This trend (not observed in the other experiments) is produced by about 1000 heterozygous variants called homozygous for the alternative allele. The cause of these calls is an unequal representation of alleles within the pool due to genetic drift, which produces a larger variance between allele read counts compared to a single heterozygous individual [see Duitama *et al.* (2014) for details].

This dataset also allowed us to test the behavior of different parameters of NGSEP (Supplementary Fig. S3). The largest effect was observed changing the minimum mapping quality score (MQ). A more stringent filtering of MQ values allowed to progressively reduce the FPPM of heterozygous SNVs without loss of sensitivity. The recent work of Langmead (2017) shows more elaborate experiments to assess how MQ values can affect variant calling. We also compared the variant calls predicted from bwa alignments (Li and Durbin, 2009) with those predicted from bowtie2 alignments (Langmead and Salzberg, 2012). In this case, variants called from bowtie2 alignments showed lower FPPM values without loss of sensitivity. Finally, allowing larger upper limits on the base quality score increased sensitivity for heterozygous variants at the cost of increased FPPM values. The opposite trend was observed for homozygous variants.

### 3.2 Human benchmark datasets

**Fig. 3.**
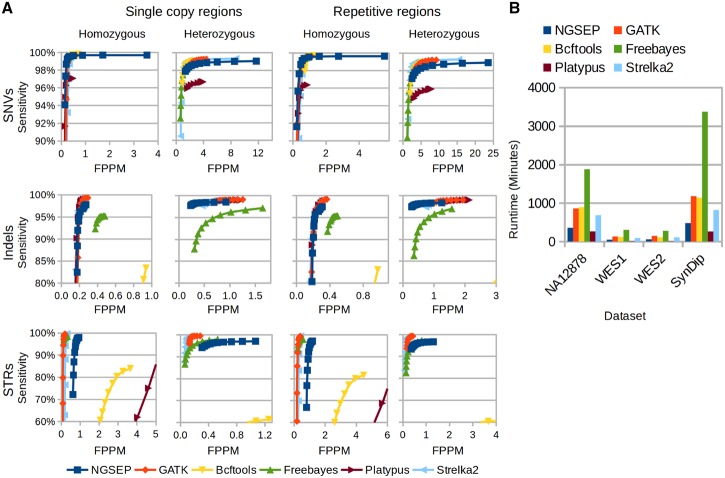
(**A**) Comparison of different tools for variants discovery from reads taken from real WGS data of the Hapmap human individual NA12878. Results are discriminated by region type (single copy or repetitive), variant type (SNVs, Indels and STRs) and genotype in the PlatGen gold standard (homozygous or heterozygous). The proportion of FPPM is used as a measure of specificity. Curves are obtained varying the filter of minimum genotype quality (GQ field in the VCF file) from 0 to 90. (**B**) Runtime in minutes taken by the six tools to analyze the evaluated human samples. WGS, WES1 and WES2 correspond to one WGS and two WES datasets taken from the human Hapmap individual NA12878. SynDip corresponds to the synthetic diploid individual developed by Li *et al.* (2018)

We analyzed whole genome sequencing reads from the Hapmap individual NA12878 for which the genome in a bottle (Zook *et al.*, 2014), and PlatGen (Eberle *et al.*, 2017) gold standards have been recently developed. The current version of PlatGen includes 1.5 million homozygous alternative variants and 2.5 million heterozygous variants. Figure 3A shows the sensitivity and FPPM achieved by each tool discriminated by variant type, gold standard genotype, repetitive context and minimum GQ filter. With the exception of Platypus for STRs and Bcftools for heterozygous indels and STRs, in this experiment all tools can achieve FPPM values below 7 for heterozygous SNVs and below 1 for homozygous variants and heterozygous indels and STRs after filtering by minimum GQ values larger than 40 (exact numbers available in the Supplementary Table S4). Before filtering, NGSEP reports FPPM values up to 25, which are produced by variant calls with one or two reads. However, these calls are effectively discarded by the minimum GQ filter with only a 0.5% loss of sensitivity. Regarding sensitivity, all tools except Platypus could call over 99% of the SNVs. For comparable FPPM values, NGSEP calls about 4000 (0.4%) less heterozygous SNVs and 1000 (0.1%) more homozygous SNVs in non-repetitive regions, compared to GATK and Bcftools. This is a good result taking into account that this benchmark has been built from combinations of genotype calls obtained using these tools. Regarding indels, Strelka2, GATK and Platypus show the best sensitivity reaching up to 98%. This sensitivity is also achieved by NGSEP for heterozygous indels but for homozygous indels it reduces to 97% due to about 1200 missing indels. Consistent with the benchmark experiment with the yeast dataset, the accuracy of Bcftools for indels and STRs and of Platypus for STRs was much lower than that of the other tools. GATK again reports larger FPPM values than NGSEP and Strelka2 for heterozygous indels. FPPM values of NGSEP are larger than those of GATK and Strelka2 for STRs. In contrast, Freebayes shows a reduction of about 5% to call indels compared to other tools and with the results obtained with the yeast dataset. Interestingly, comparable sensitivity and FPPM values are observed between non-repetitive and repetitive regions. An increase in FPPM values is only observed for heterozygous SNVs. This outcome is also observed in experiments with the SynDip benchmark dataset (see below) and with simulated reads from the rice reference genome (Supplementary Text) and suggests that misalignments within or close to STRs are more important as a source of errors than incorrect mapping produced by large repetitive structures.

We also re-analyzed WES data from two different capture experiments from DNA of NA12878 (termed WES1 and WES2), available at the sequence read archive database (Supplementary Table S2). For WES1, sequenced using the SeqCap capture technology, sensitivity decreases about 5% for SNVs, 10% for indels and up to 30% for STRs, compared to that obtained from WGS data within the same regions (Supplementary Fig. S4). This outcome can be explained by the capture efficiency (about 82%). FPPM values were at least two times larger than those obtained from WGS data in non-repetitive regions. The most extreme case is observed for heterozygous SNVs called by Strelka2 with an increase of FPPM up to 200. FPPM values of NGSEP only increase more than two times for low quality homozygous SNVs and for heterozygous STRs (Supplementary Table S4). This makes NGSEP outperform Strelka2, Platypus and GATK for heterozygous indels in this experiment. Regarding WES2, the reduction in sensitivity and increase of FPPM values is much larger than that observed with WES1 (Supplementary Fig. S5), presumably due to a shorter read length (76 versus 100 bp), lower sequencing effort (8.88 versus 12.69 gbp), and lower capture efficiency (52 versus 82%). Intersecting TruSeq capture regions with exons fully covered by reads taken from this experiment the sensitivity is restored but FPPM values of heterozygous variants remain larger than those calculated from WGS genotype calls within the same regions.

We further analyzed reads from the recently developed synthetic diploid genome (Li *et al.*, 2018). The main difference observed in this benchmark compared to the PlatGen benchmark dataset was an important increase in FPPM for all methods in heterozygous variants (Supplementary Fig. S6) despite of the higher average read depth of this dataset (48 versus 32×). This reflects the possible biases introduced in the construction of the PlatGen benchmark and the fact that the PlatGen benchmark has been used for quality improvement in the development of most of the tools. Sensitivity is also reduced by 5% for indels and by 20% for STRs, especially in repetitive regions (Supplementary Table S5). Strelka2 and GATK are more accurate than NGSEP for indels and STRs in this comparison, probably because this dataset was used as reference to improve quality in the latest versions of these tools. NGSEP is more accurate than Strelka2 for homozygous SNVs and more accurate than GATK for heterozygous SNVs in non-repetitive regions.

Finally, Figure 3B shows the comparison of runtimes to analyze the different datasets of human WGS and WES. Platypus was consistently the most efficient tool followed by NGSEP. GATK and Freebayes were the least efficient being between 2 and 4 times slower than NGSEP. This trend was consistent over species and sequencing protocols (Supplementary Table S6).

### 3.3 Biparental populations of rice and cassava

**Fig. 4.**
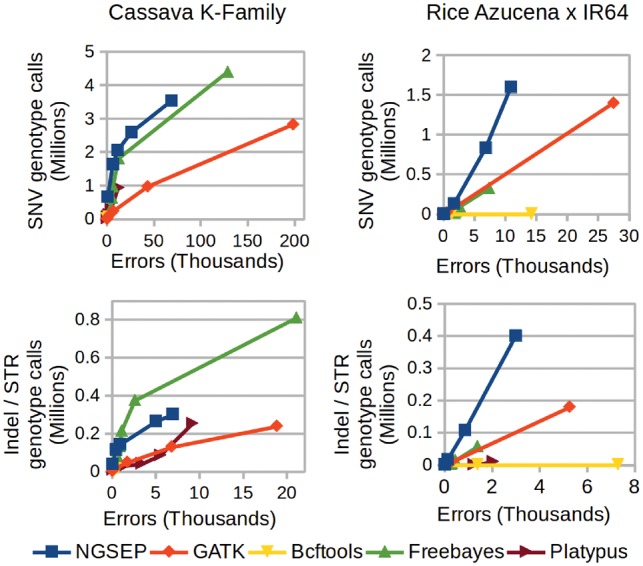
Comparison of different tools for population variants discovery and genotyping using reads taken from real GBS experiments on two different biparental populations of cassava (left panels) and rice (right panels). In absence of a gold standard, the total number of genotype calls is used as a measure of sensitivity and the number of errors inferred from the population structure is used as a measure of specificity. Curves are obtained varying the filter of minimum genotype quality (GQ field in the VCF file) according to the observed distribution of GQ values for each tool

To validate the efficiency and accuracy of the different variant callers on populations, we re-analyzed GBS data for two biparental breeding populations of cassava and rice for which the distribution of allele and genotype frequencies can be predicted by structure and inheritance rules. Whereas the cassava K-family is an F1 population (Soto *et al.*, 2015), the rice population is an F6 with low expected levels of heterozygosity due to inbreeding (Spindel *et al.*, 2013). Because both populations have more than 100 individuals, accurate predictions of both allele and genotype frequencies could be derived regardless of individual genotype errors and inconsistent genotype calls can be identified. Figure 4 shows the number of genotype calls that could be obtained using the different tools on both populations as a function of the number of genotyping errors. NGSEP and Freebayes consistently produce better results than GATK, Bcftools and Platypus on SNVs, indels and STRs. Platypus and GATK show less sensitivity for similar numbers of errors compared to the other tools, probably due to the ineffectiveness of the local assembly algorithm for GBS data. Freebayes outperforms NGSEP for indels but NGSEP is more accurate for SNVs. The lower performance of GATK is not consistent with our previous benchmark in which GATK 3 had a similar accuracy than NGSEP (Perea *et al.*, 2016). Recent changes in the algorithm and software parameters implemented in GATK 4 to increase accuracy in human datasets could explain this outcome. For the case of the rice population NGSEP more clearly outperforms other software tools, including Freebayes. Our previous benchmark experiment with an also inbred bean population (Perea *et al.*, 2016) produced similar results. The rice population analyzed in this work was chosen over the bean population because the number of individuals is larger, the population is biparental and the parents are included. However, the effort to sequence this population was much lower than that of the cassava population and this impacted the distribution of quality scores (GQ) for all tools (Supplementary Fig. S7). Only NGSEP could achieve close to one million SNV genotype calls with less than seven thousand errors using 35 as minimum GQ value. We speculate that this outcome is produced by the assumption of Hardy–Weinberg equilibrium that is assumed by other models (Li, 2011), but is clearly violated by advanced populations of autogamous species such as rice. In contrast, the accuracy of Freebayes becomes similar to that of NGSEP for the cassava population because cassava is an allogamous species and hence most of the variants in an F1 should be in Hardy–Weinberg equilibrium.

## 4 Discussion

Accurate variant genotyping is a building block of current pipelines for genetics based medicine. It is also a critical procedure for genome-wide association studies and genomic prediction pipelines in plant breeding. Hence, continuous quality assurance and sustainability is critical for tools performing variants detection and genotyping. Here we report the new algorithms implemented in NGSEP and provide an updated comparison with the tools currently used by different research groups in a wide range of experimental designs and species. Comparisons performed on both the human PlatGen dataset (Eberle *et al.*, 2017) and the SynDip dataset (Li *et al.*, 2018) show that NGSEP is competitive in accuracy compared to approaches based on mini-assembly (Kim *et al.*, 2018; Poplin *et al.*, 2017; Rimmer *et al.*, 2014) within both repetitive and non-repetitive regions. NGSEP also shows better accuracy for analysis of GBS data from populations of two different plant species. It has been already noted that approaches based on mini-assembly are not effective to increase accuracy on amplicon sequencing (Yang *et al.*, 2015). Our results indicate that this would also be the case for GBS data because the reads in this case are not randomly taken from different overlapping regions but are stacked in discrete loci. Regarding efficiency, only Platypus was consistently faster than NGSEP but this comes at the cost of reduced accuracy, even in the comparisons with human gold standard datasets. Using the yeast benchmark dataset we also assessed the performance of DeepVariant, which is a recently published solution based on a convolutional neural network (Poplin *et al.*, 2018). The outcome of this assessment was a consistent reduction of FPPM values compared to previous solutions, however at the cost of reduced sensitivity for SNVs and STRs. The runtime of this tool was two times larger than that of GATK and it took close to four times the RAM needed by other tools to analyze the yeast unselected pool. Regardless of the particular ranking achieved by each tool on each benchmark dataset, the comparisons presented in this manuscript are only a snapshot in the evolution of each software package across time. During the development of this work we have witnessed quality improvements performed by the developers of each tool. The improvement achieved by the development of the algorithms described in this manuscript is evidenced by the lower performance of a previous version of NGSEP in the comparison using the yeast benchmark dataset. Based on our experience with NGSEP users, we believe that the accuracy and efficiency achieved by NGSEP, plus the implemented usability features including a rich graphical interface and integration with web-based frameworks, substantially increases the number of research groups that are able to carry on experiments involving HTS, thus contributing to the democratization of this technology.

The confounding effect of misplaced reads within repetitive structures has been considered a major issue for variants detection from short reads (Li and Wren, 2014). Although comparisons with the human benchmark datasets show up to two times larger FPPM in repetitive regions compared to single copy regions, these differences are much smaller than our initial expectations. A possible explanation for this outcome could be that current paired-end reads span enough mutations between copies of most repetitive structures, increasing the likelihood of correct read alignment. Conversely, read misalignment around STRs remains a major source of errors for variants detection and genotyping. In contrast with approaches based on mini-assembly, our solution performs a single realignment and haplotype clustering for each complete STR structure. This informed approach leads to comparable accuracy and higher efficiency than that of methods based on mini-assembly, taking into account that assembly based on deBruijn graphs tends to be cumbersome in repetitive regions. Our approach also leads to a more informative annotation of the genomic context of each variant. The cost of this approach is a reduction of sensitivity for genotyping of the STRs themselves because NGSEP explicitly excludes from the analysis STRs having a total length longer than the read length.

The importance of variants detection and genotyping in different fields can be demonstrated by the large number of benchmark studies recently reported by different groups (Supplementary Table S1). Some of these works report surprisingly low values of accuracy, even for widely used analysis pipelines (Sandmann *et al.*, 2017). To understand this issue, we performed a qualitative assessment of these studies evaluating if and how they discuss different aspects of variant detection and genotyping. We found different issues including lack of a gold standard set of genotypes (Ghoneim *et al.*, 2014), lack of information about parameters used for each tool and comparison procedures (Bao *et al.*, 2014), overlooking of predicted and gold standard genotype calls beyond variants discovery (Hasan *et al.*, 2015; Ribeiro *et al.*, 2015), and validation limited to a small number of variants genotyped by Sanger sequencing, usually avoiding difficult regions (Kim *et al.*, 2017). Some benchmarks also fail to use appropriate parameters for the evaluated use cases such as variants discovery in pooled exome sequencing data (Hasan *et al.*, 2015; Sandmann *et al.*, 2017). Other benchmark experiments were well conducted but limited to a particular type of variant (Fang *et al.*, 2014; Korneliussen *et al.*, 2014) or genomic region. The review performed in Tian *et al.* (2016) focuses on variants discovery from WES data in regions of high diversity within the human genome such as the major histocompatibility complex. Other reviews focus on the detection of somatic and cancer mutations from WES data (Hofmann *et al.*, 2017; Xu, 2018). As expected, most of the studies focus on human samples. As an exception, in Ribeiro *et al.* (2015) the authors perform simulation experiments from the arabidopsis genome. However, the simulations only include negative cases and hence only false positive rates are estimated. The benchmark experiments presented in this manuscript aim to provide updated accuracy information on methods able to detect SNVs, indels and STRs from reads aligned to a reference genome. We also take into account the, usually overlooked, genotype quality scores reported by the different tools according to their underlying models for variant calling. Understanding that accurate genotyping is as important as variant detection for different applications, the comparisons presented in this work also evaluate the capacity of the different tools to perform accurate genotyping discriminating gold standard homozygous and heterozygous genotypes. To the best of our knowledge, this work is the most complete assessment of accuracy of variant detection and genotyping tools in the widest range of species, sequencing protocols and experimental settings.

Regarding indels and STRs, important differences are observed in previous evaluations, sometimes due to methodological issues but also because it is not straightforward to assess if a predicted indel call matches a gold standard call (Sun and Medvedev, 2017). Depending on the strategy for local alignment, a correct indel call can be counted as a false positive because the initial position does not match. Only a small fraction of previous works discuss this issue and provide details on how the comparisons are performed (Fang *et al.*, 2014; Li and Wren, 2014). Moreover, it has been shown that STRs have a much higher mutation rate compared to SNVs and that variation in STRs is causative for different traits and diseases in different species (Gemayel *et al.*, 2010). Unfortunately, most benchmark datasets overlook this type of variation or just use STR annotations as a filtering procedure (Eberle *et al.*, 2017; Li *et al.*, 2018). This complicates the exact quantification of sensitivity and specificity of the different tools within these regions. Taking into account this scenario, the recent work of Sun and Medvedev (2017) shows that gold standard and test variant matching can be in itself an interesting bioinformatics problem and presents the software tool VarMatch, which tries to address this problem predicting at the same time locations of STRs. We developed and integrated in NGSEP our own evaluation procedure which follows a strategy similar to VarMatch but uses predictions of STR locations from tools such as tandem repeats finder (Benson, 1999) and takes into account that current gold standard datasets are already phased. Our validation procedure reconstructs the benchmark haplotypes for each STR based on the phased gold standard variants and then matches together the variants called within the region to check if the correct haplotypes could be inferred from the called variants. Although in principle this solves the issue of not having STR annotations in the human benchmark datasets, we believe that future construction of gold standard genomes should include a clear annotation, genotyping and phasing of STRs to facilitate variant calling evaluation in these regions.

Different versions of NGSEP have been used already by several different research groups working in a wide range of species including yeast, leishmania, rice, common bean, lima bean, cassava and other species. We plan to keep evolving NGSEP looking for improved algorithms for variants detection and also adding new functionalities for variants interpretation, samples clustering and other downstream analyses of genomic variation datasets. We expect that the results presented in this work will contribute to the continuous improvement of accuracy and efficiency in variant calling needed to achieve the quality required for personalized medicine and for genomic selection in plant and animal breeding.

## Supplementary Material

btz275_Supplementary_Materials
