# Supplementary material for: NGSEP3: accurate variant calling across species and sequencing protocols
Source: Bioinformatics. 2019 Apr 25;35(22):4716–23. doi: 10.1093/bioinformatics/btz275 (PMC6853766; doi:10.1093/bioinformatics/btz275)

Supplementary material for:

NGSEP3: Accurate variant calling across species and sequencing protocols

Daniel Tello[1,†] , Juanita Gil[1,†] , Cristian D. Loaiza[2,‡] , John J. Riascos[2] , Nicolás Cardozo[1] and Jorge Duitama[1,3,*]

1. Systems and Computing Engineering Department, Universidad de los Andes, Bogotá, Colombia
2. Centro de Investigación de la caña de azúcar de Colombia, CENICAÑA, Cali, Colombia
3. Agrobiodiversity Research Area, International Center for Tropical Agriculture, Cali, Colombia
† Co-first authors
‡ Present address: Department of Plants, Soils, and Climate. Utah State University, Logan, Utah, USA

* To whom correspondance should be addressed. ja.duitama@uniandes.edu.co

Supplementary text

Read alignment

Raw reads from the biparental populations were demultiplexed using the Demultiplex command of NGSEP. Reads from all samples were aligned to their corresponding reference genome using bwa 0.7.17 (Li and Durbin, 2009). Yeast samples were also independently aligned using bowtie2 2.3.4.1 (Langmead and Salzberg, 2012). Alignments were sorted by reference coordinates using picard [https://broadinstitute.github.io/picard/]. This tool was also used to merge the read alignments from the individuals within the GBS populations. Catalogs of Short Tandem Repeats (STRs) for each species were obtained from their corresponding reference genomes running the tool tandem repeats finder (Benson, 1999) using the parameters recommended in their manual.

Details of parameters to execute the tools evaluated in this study

NGSEP: The command FindVariants of version 3.3.1 was used for the different experiments with WGS and WES data described in this manuscript. Calibration of parameters was performed with both the yeast real data and with the simulated data (see below). The values 2, 3, 4 and 5 were tried for the maximum number of reads starting at the same position (option -maxAlnsPerStartPos). The minimum mapping quality to call an alignment unique (option -minMQ) was also varied taking the values 0, 10, 20, 30 and 40. The parameter for maximum base pair quality score (-maxBaseQS) was also varied taking the values 10, 20, 30, and 40. Options for considering only unique, primary or secondary alignments were also compared (options -p and -s). Based on these calibration experiments, comparison with other tools in the yeast and human datasets was performed using the options "-maxAlnsPerStartPos 2 -maxBaseQS 30 -minMQ 40" and with the option -knownSTRs to load a catalog of known short tandem repeats for each sample. For the GBS populations, the command MultisampleVariantsDetector was used with the options "-maxAlnsPerStartPos 100 -maxBaseQS 30 -minMQ 40" and with the option -knownSTRs to load a catalog of known short tandem repeats for each sample.

GATK: To run the Genome Analysis Toolkit (GATK-v4.1.0.0) it was necessary to generate a Sequence Dictionary from the reference file with picard. For the variants discovery step, the HaplotypeCaller module was used with genotyping mode set to DISCOVERY. The minimum phred-scaled confidence

threshold was set to 0 to obtain results with maximal sensitivity and evaluate the performance of this tool on different minimum GQ values. For GBS data of rice and cassava populations, the parameter "--max-reads-per-alignment-start" was set to 100 to match the value of the corresponding parameter "-maxAlnsPerStartPos" of NGSEP.

Bcftools: Version 1.9 was executed in two steps: first the mpileup process was run activating the option annotate, enabling fields DP and AD in the VCF file; then the call option was run activating the multiallelic-caller option and reporting variant sites only. The format field GQ was also enabled to evaluate the performance of this tool on different minimum GQ values.

Freebayes: Version v1.2.0-4-gd15209e was run for all the samples activating the parameters "-0 --genotype-qualities --strict-vcf", to obtain GQ values for the genotype calls and evaluate the performance of this tool on different minimum GQ values.

Platypus: Version 0.8 was executed with default parameters using the option callVariants.

Strelka: Version 2.9.12 was executed following the instructions in the manual, running first the script "configureStrelkaGermlineWorkflow.py" with default parameters and then the script "runWorkflow.py" with the options -m local -j 1 -g 8.

DeepVariant: V0.7 was executed with default parameters using the instructions to load a Docker image explained in their quick start guide (https://github.com/google/deepvariant/blob/r0.7/docs/deepvariant-quick-start.md).

Simulation experiments

Simulation experiments were performed to assess the efficiency and accuracy of NGSEP in a large number of scenarios including changes in average read depth and genomes with a larger percentage of repetitive regions. We built a functionality within NGSEP to simulate individuals based on a reference genome with different ploidies and different types of variation (SNVs, small indels and variable STRs). This functionality can be executed in command line mode (SingleIndividualSimulator command) or through the graphical interface. The script generates a fasta file with the simulated genome sequence and a genomic VCF file with the reference locations, alternative alleles and gold standard genotypes of the simulated variants. We simulated diploid individuals using as baseline the rice reference genome. Variants were simulated using SNV and indel rates of 0.001 and 0.0001 respectively and 0.1 as the fraction of STRs with non-reference alleles. Gold standard genotypes were assigned randomly as homozygous or heterozygous. Paired end reads with average fragment length of 500 bp, read length of 200 bp, error rate of 0.01 and average read depth of 5x, 10x, 20x, 30x and 50x were simulated for each simulated individual using the wgsim software package [https://github.com/lh3/wgsim], setting the options -r, -R and -X to zero to avoid further simulation of variants by this tool.

The size of the rice genome is 400Mbp and, although is much smaller than the human reference genome, it has an approximate repetitive content of 50%, which makes it adequate to evaluate the behavior of each tool in both repetitive and non-repetitive regions. Supplementary figure 8 shows ROC like curves obtained varying the filter of minimum genotype quality. In most scenarios NGSEP provides comparable accuracy compared to other tools, closely followed by the GATK haplotype caller and Strelka2. These tools only achieved better accuracy for heterozygous STRs in repetitive regions. The FPPM was about 10 times larger for heterozygous than for homozygous calls and about 2 times larger for repetitive regions than for single copy regions. Consistent with the results obtained from the

real datasets, Bcftools shows a much larger FPPM than the other tools in heterozygous indels and STRs and Platypus also shows less accuracy for STRs. FPPM values seem to be larger for SNVs than for indels and STRs. In principle the cause of this outcome is that only substitution errors were simulated. However, this pattern is also observed in real data, probably because the indel error rates of Illumina sequencing are much smaller than the substitution error rates.

Simulations were also performed from the yeast reference genome using different combinations of ploidy (1, 2, 3 and 4), and average read depth (5x, 10x, 20x, 30x, and 50x) to evaluate the behavior of each tool under these conditions. Using a minimum genotype quality score of 10, the sensitivity reduced up to 40% for heterozygous sites at 5x read depth (Supplementary figure 9). Except for Freebayes, all tools recovered a sensitivity over 80% at 10x and 95% at 20x. Regarding ploidy, all tools showed reduced sensitivity for triploids and tetraploids but the reduction was more pronounced for GATK and Freebayes which showed sensitivity below 40% for tetraploids, whereas for the other tools the sensitivity only reduced to 80%.

Efficiency and other software quality attributes

We measured and compared the running time of the different tools on the different datasets presented in this study. NGSEP was consistently the second best efficient tool (Supplementary table 6). Only Platypus reported smaller runtimes than NGSEP. Bcftools ranked third being faster than NGSEP only for the rice population (however with very poor results). For individual sample cases Bcftools was up to 2 times slower than NGSEP and for the cassava population it was 2.5 times slower. Even using 4 cores per process, GATK was between 2.3 and 16 times slower than NGSEP for single individuals (not counting marking of duplicates and quality score recalibration) and about 5 times slower for the rice population. Freebayes was faster than GATK for the yeast Unselected pool but ranked last in the other datasets.

Regarding other software quality attributes, NGSEP is the only tool that can be fully operated from a graphical interface. Similar to other tools, NGSEP can be included in a web integration pipeline such as Galaxy (Goecks et al., 2010). The parameters included in NGSEP allow to work directly with results generated from the read alignment tools (sorted by reference coordinates) avoiding the time and disk space consuming steps of duplicate marking or quality score recalibration required by other pipelines. All tools produce their output in the standard variant call format (VCF) which facilitates integration with downstream analysis tools. However, Freebayes does not provide the genotype quality value (GQ) by default and if the option is activated, the GQ is a real number and not an integer as defined in the specification. This tool also fails to provide likelihoods for ploidy greater than 2. The VCF file provided by Platypus is not sorted by reference sequence so, for species different than human it needs to be resorted. Consistent with the results reported by (Kim et al., 2018) Strelka2 seems to be more accurate and efficient than the GATK haplotype caller. However, it only works with paired-end data and then it could not be tested with the GBS population datasets.

Supplementary tables

Supplementary table 1. Survey of studies reporting new tools and pipelines for variants detection, benchmark datasets and comparisons between tools (Separate Excel file).

Supplementary table 2. Benchmark datasets used in this study (Separate Excel file).

Supplementary table 3. Sensitivity and FPPM values for experiments with the benchmark yeast unselected pool (Separate Excel file).

Supplementary table 4. Sensitivity and FPPM values for experiments with WGS and WES data of the NA12878 Hapmap human individual compared to the PlatGen gold standard set of genotype calls (Separate Excel file).

Supplementary table 5. Sensitivity and FPPM values for experiments with WGS data from the synthetic diploid (SynDip) human dataset developed by Li et al., 2018 (Separate Excel file).

Supplementary table 6. Runtime of each software tool on each benchmark dataset (Separate Excel file).

Supplementary figures

Supplementary figure 1. Example in which a misalignment of reads partially spanning a homozygous STR can produce a false heterozygous genotype call and possibly a false SNV call.

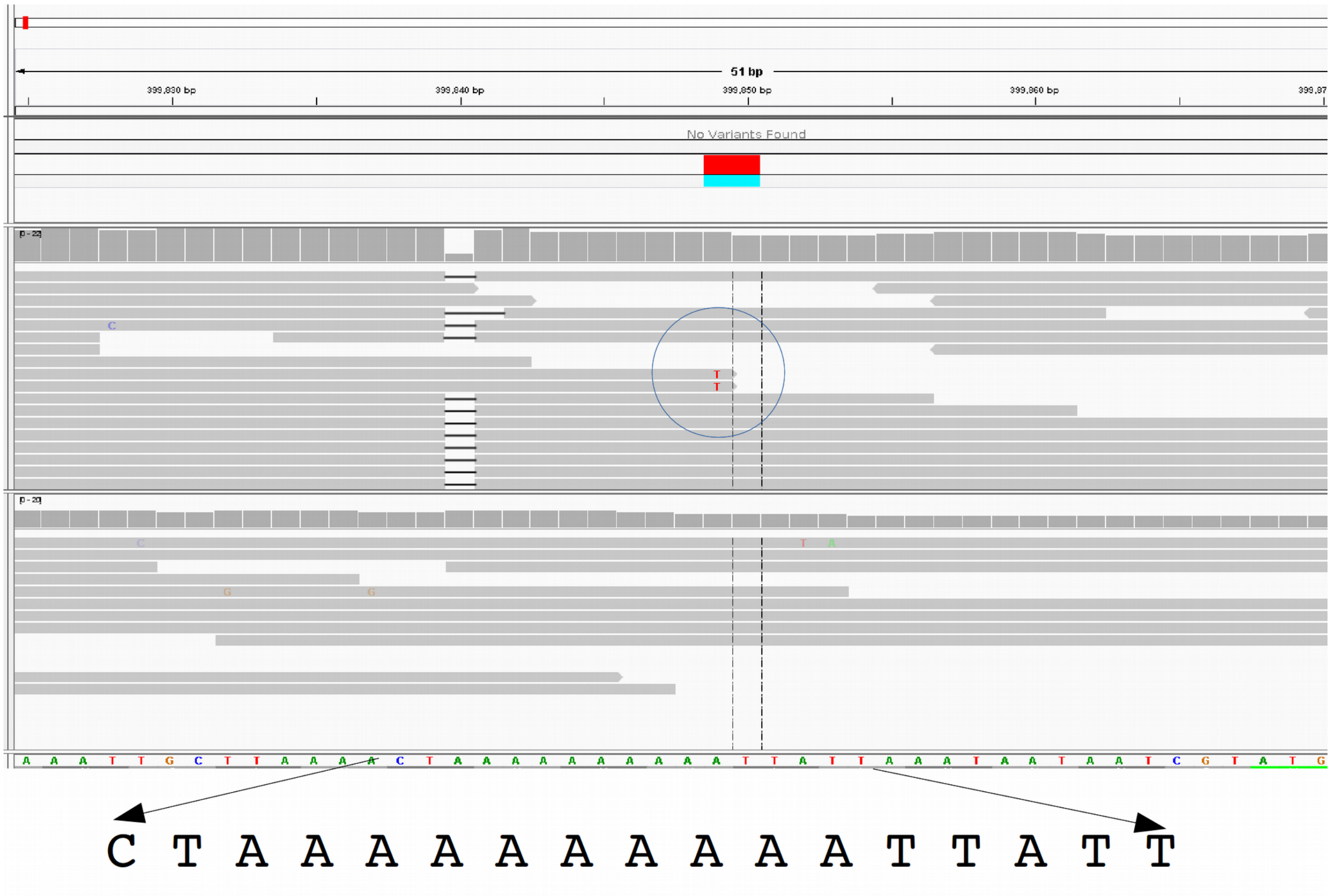

Supplementary figure 2. Comparison of different tools for variants discovery from reads taken from a F1 pool of segregants derived from two yeast haploid strains. Results are discriminated by variant type (SNVs, Indels and STRs) and gold standard genotype (homozygous variant or heterozygous). The proportion of false positives per million basepairs (FPPM) is used as a measure of specificity. Curves are obtained varying the filter of minimum genotype quality (GQ field in the VCF file) from 0 to 90.

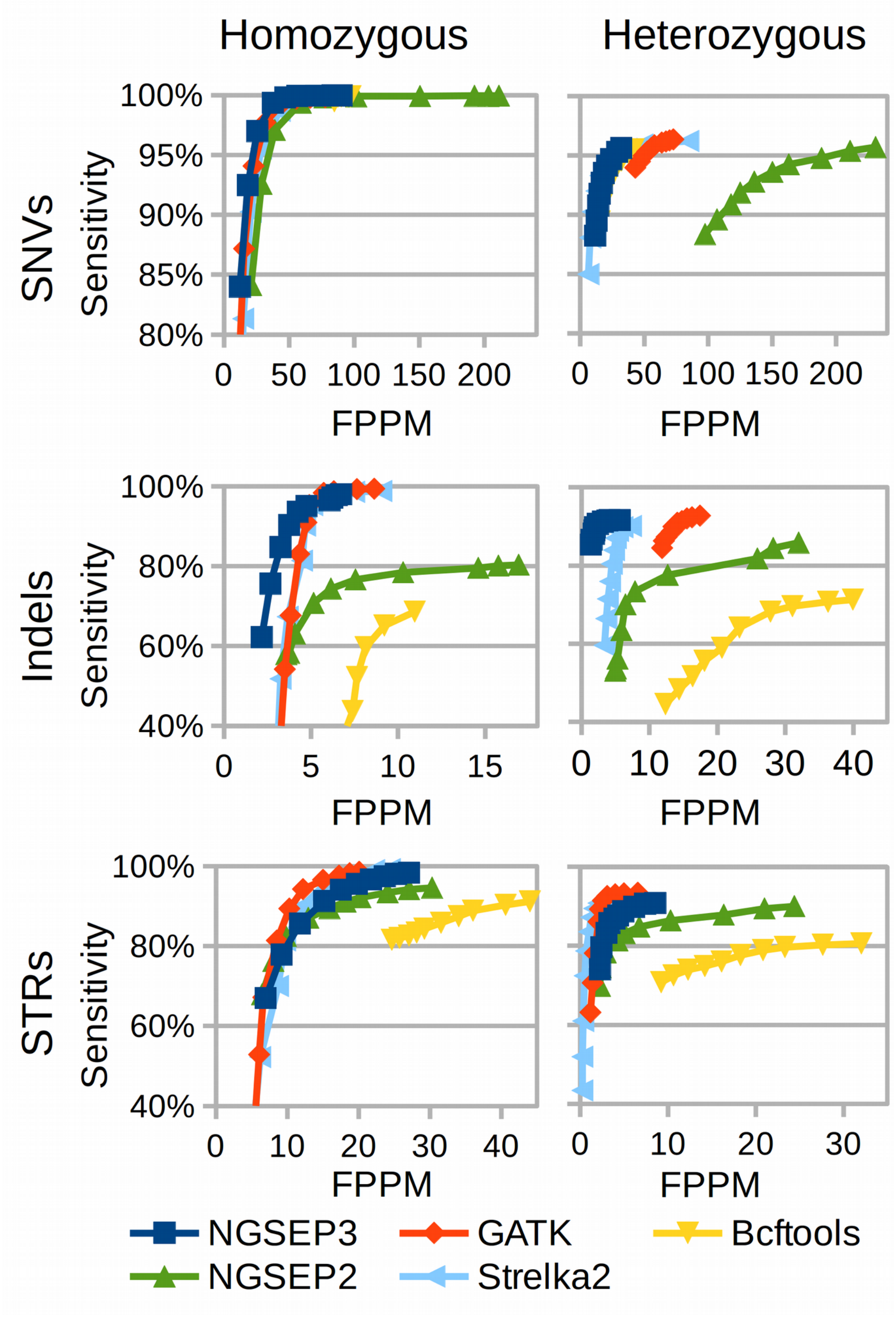

Supplementary figure 3. Behavior of NGSEP for SNVs under A) different values of the minimum mapping quality for bwa alignments, B) different read alignment tools, and C) different values of the maximum base quality score.

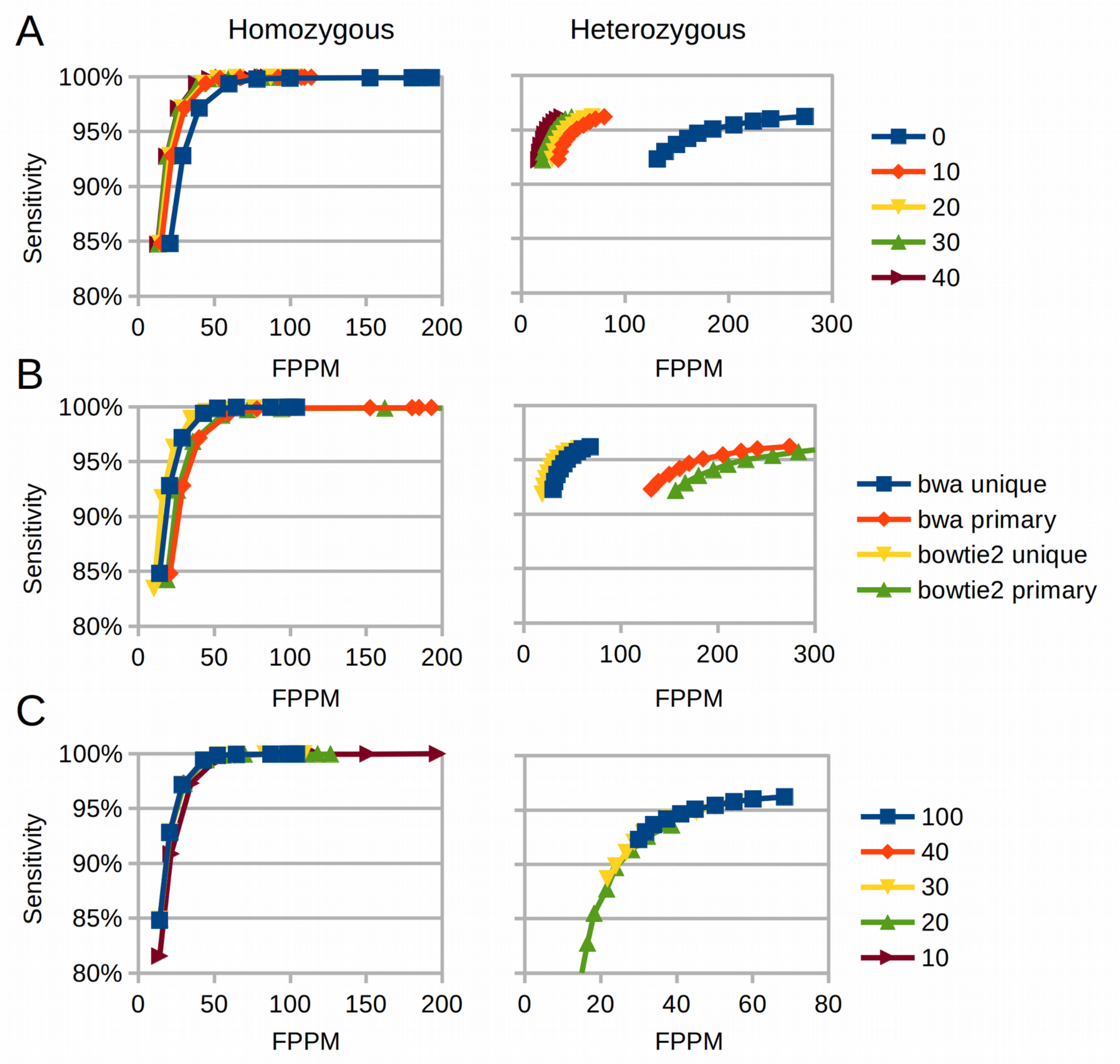

Supplementary figure 4. Comparison of different tools for variants discovery from reads taken from real Whole Exome Sequencing (WES) data of the Hapmap human individual NA12878 (WES1 dataset). Results are discriminated by variant type (SNVs, Indels and STRs) and genotype in the Platinum genomes gold standard (homozygous variant or heterozygous). The proportion of false positives per million basepairs (FPPM) is used as a measure of specificity. Curves are obtained varying the filter of minimum genotype quality (GQ field in the VCF file) from 0 to 90. Results obtained from WES data are compared with those obtained from WGS data in the regions captured by the SeqCap protocol.

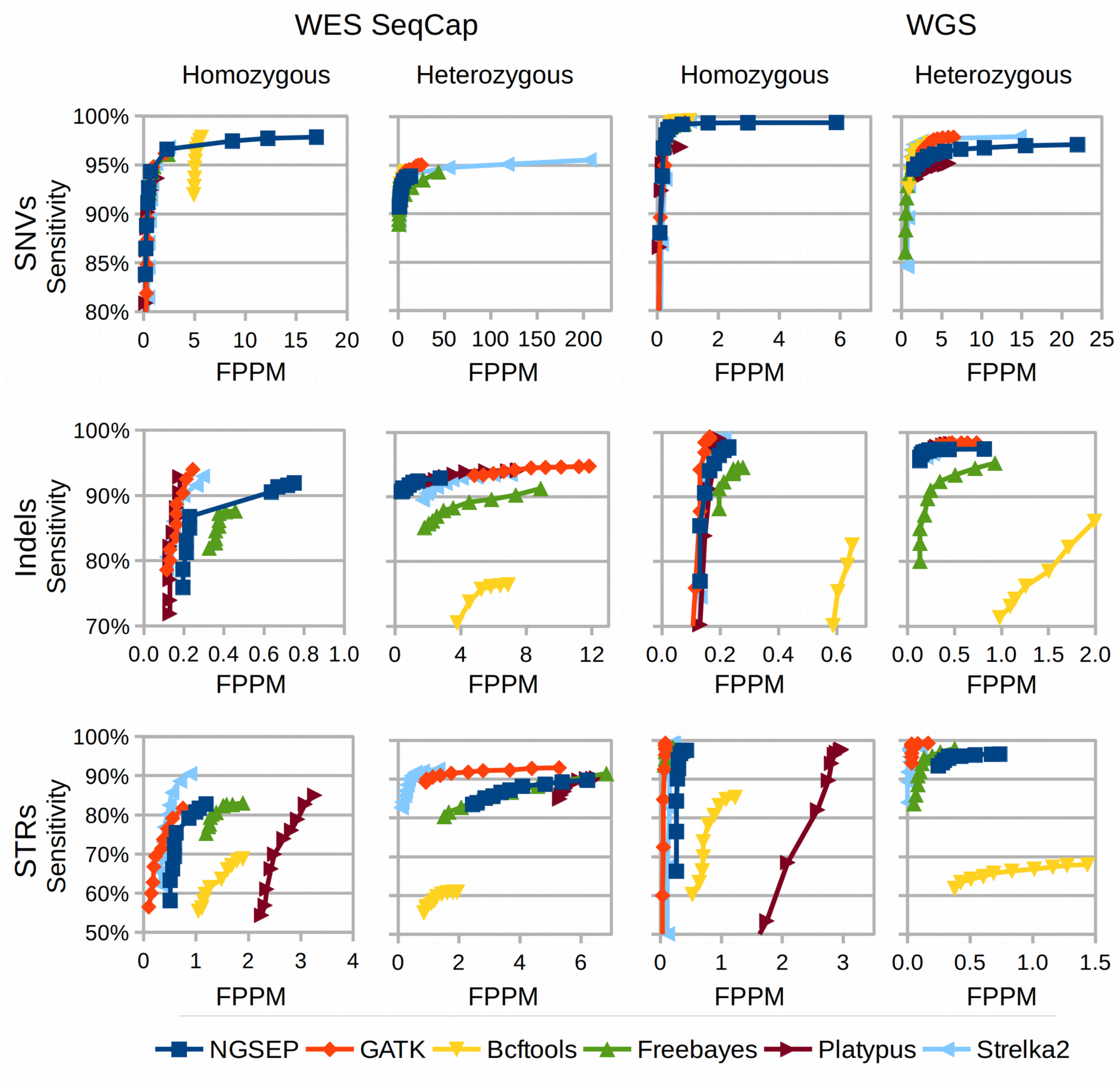

Supplementary figure 5. Comparison of different tools for SNV discovery from reads taken from real WES data of the Hapmap human individual NA12878 (WES2 dataset). Results are discriminated by genotype in the Platinum genomes gold standard (homozygous variant or heterozygous). The proportion of false positives per million basepairs (FPPM) is used as a measure of specificity. Curves are obtained varying the filter of minimum genotype quality (GQ field in the VCF file) from 0 to 90. Results obtained from WES data are compared with those obtained from WGS data in the regions captured by the TruSeq protocol (top panel) and in the intersection of regions captured by the TruSeq protocol and the exons fully covered by WES data (bottom panel).

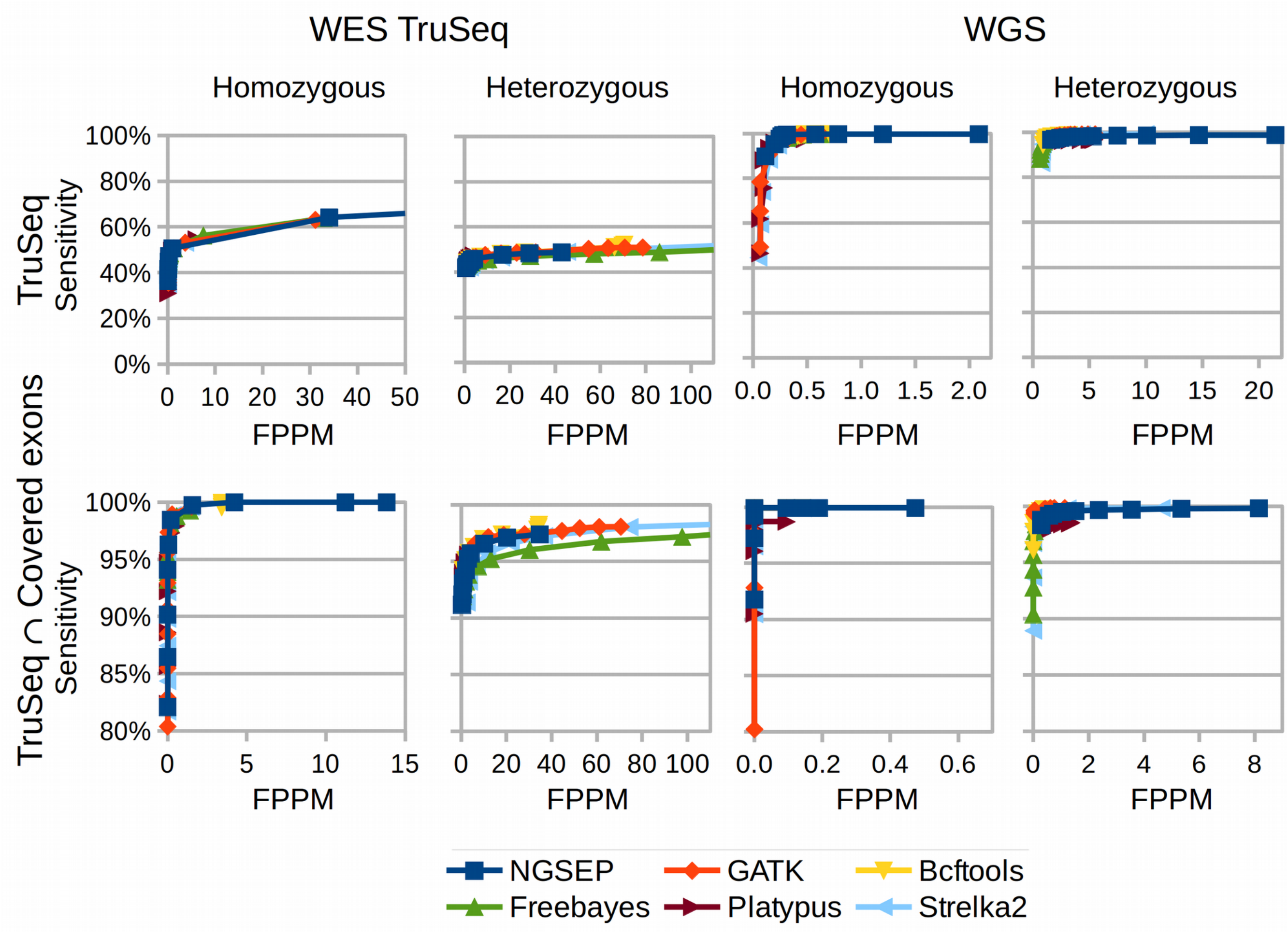

Supplementary figure 6. Comparison of different tools for variants discovery from reads taken from real WGS data of the synthetic diploid individual developed by Li et al. 2018. Results are discriminated by region type (single copy or repetitive), variant type (SNVs, Indels and STRs) and genotype in the SynDip gold standard (homozygous variant or heterozygous). The proportion of false positives per million basepairs (FPPM) is used as a measure of specificity. Curves are obtained varying the filter of minimum genotype quality (GQ field in the VCF file) from 0 to 90. Results for indels and STRs for Bcftools are not shown because the sensitivity of this tool was below 80% for indels and below 60% for STRs.

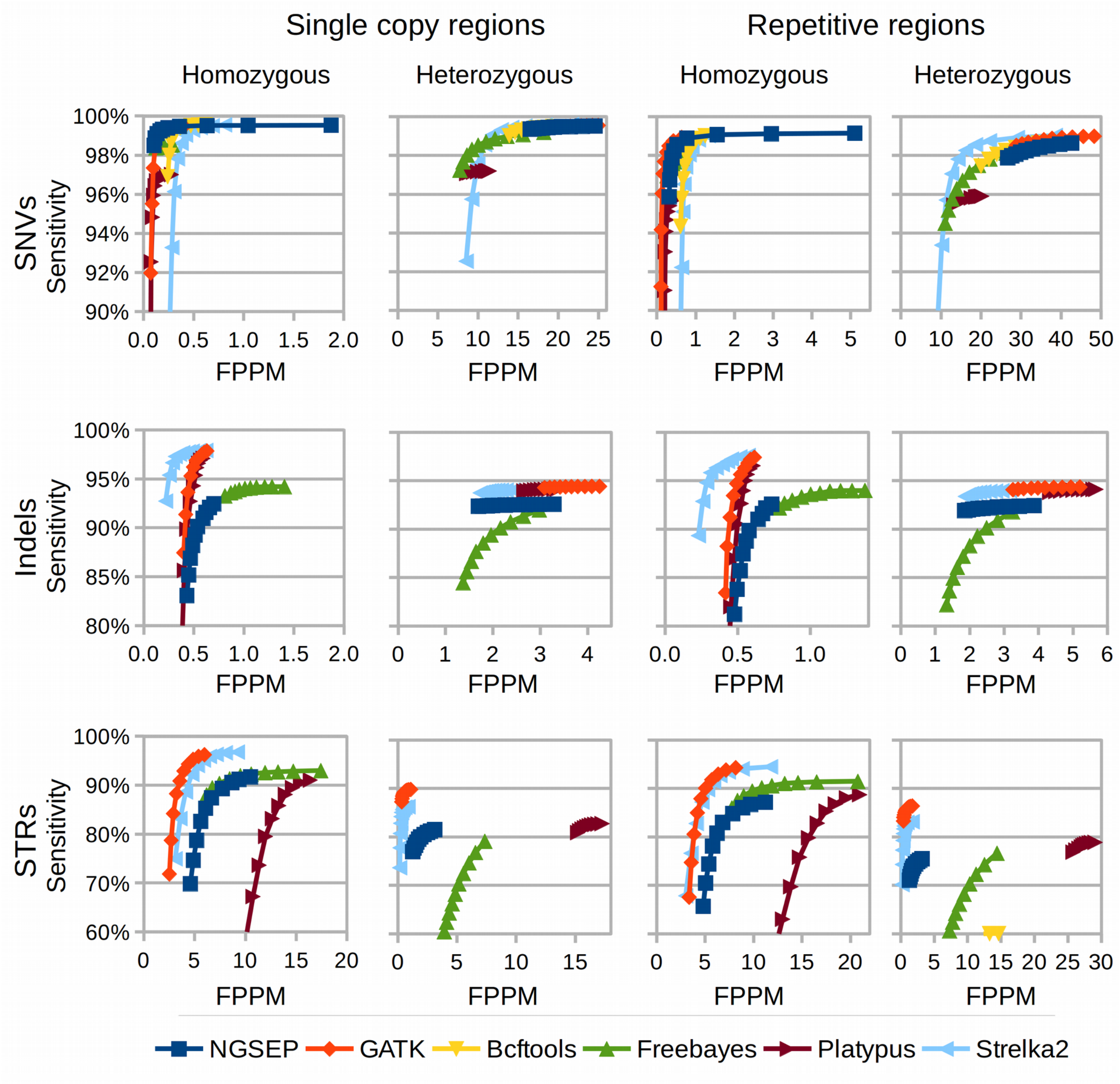

Supplementary figure 7. A. Distribution of read depth per datapoint in variant sites called by NGSEP on the cassava and rice biparental populations. B. Percentage of datapoints at different values of the minimum genotype quality score for variants called on the cassava biparental population. C. Percentage of datapoints at different values of the minimum genotype quality score for variants called on the rice biparental population.

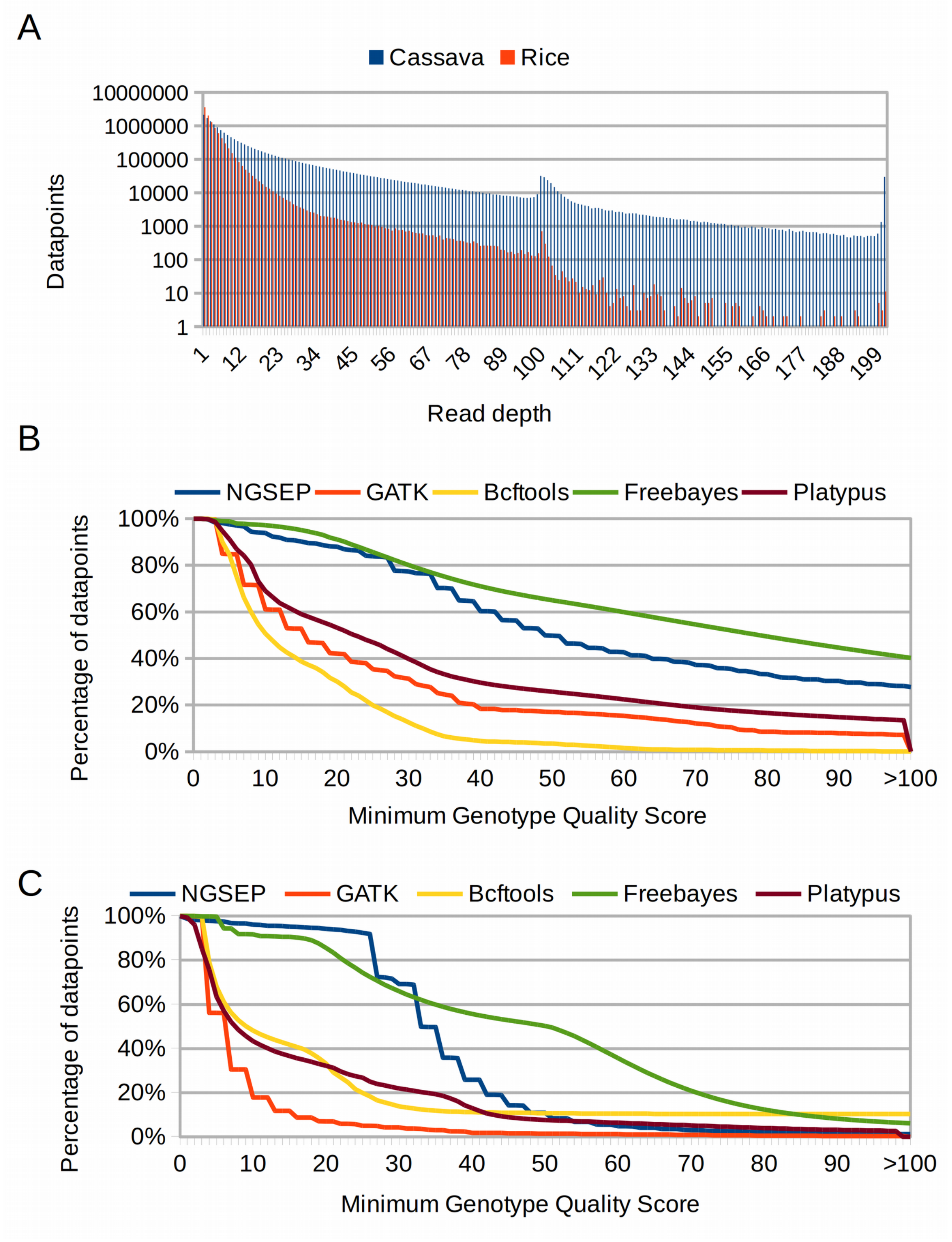

Supplementary figure 8. Comparison of different tools for variants discovery from simulated reads from a simulated rice diploid individual. Results are discriminated by region type (single copy or repetitive), variant type (SNVs, Indels and STRs) and genotype in the simulated gold standard (homozygous variant or heterozygous). The proportion of false positives per million basepairs (FPPM) is used as a measure of specificity. Curves are obtained varying the filter of minimum genotype quality (GQ field in the VCF file) from 0 to 90. Results for homozygous indels and STRs for Bcftools are not shown because the FPPM of this tool was above 5 for indels and above 15 for STRs.

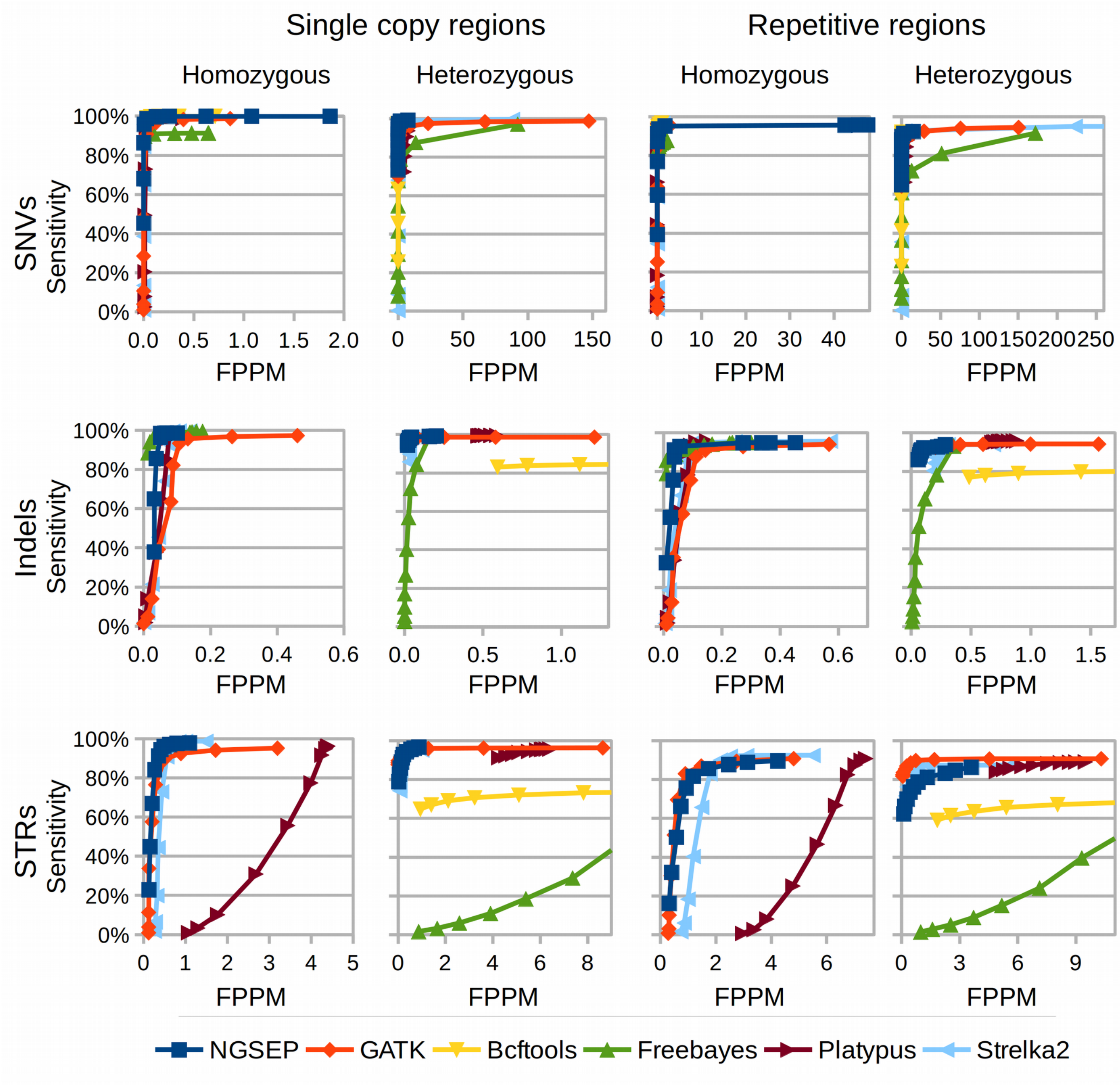

Supplementary figure 9. Comparison of different tools for variants discovery for simulations of individuals from the yeast reference genome varying average read depth and normal ploidy. The F-score is calculated as the harmonic mean of precision and recall.

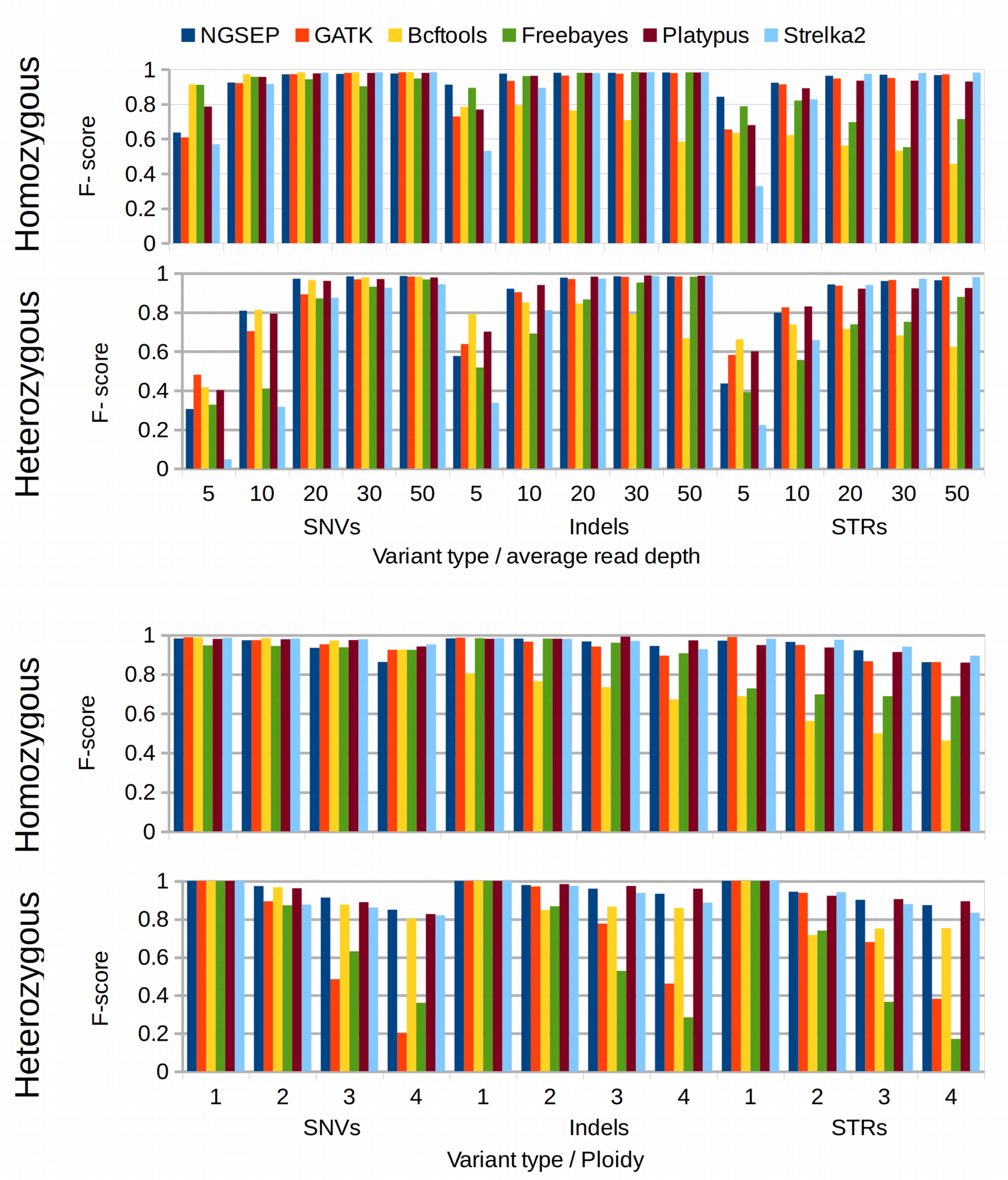

Supplement: btz275_Supplementary_Materials [file btz275_supplementary_materials.zip › btz275-suppl_data/SupplementaryMaterials.pdf]
